# Progress and Trends of Optical Microfiber-Based Biosensors

**DOI:** 10.3390/bios13020270

**Published:** 2023-02-14

**Authors:** Yasmin Mustapha Kamil, Muhammad Hafiz Abu Bakar, Nurul Hida Zainuddin, Mohd Hanif Yaacob, Mohd Adzir Mahdi

**Affiliations:** Wireless and Photonic Networks Research Centre, Faculty of Engineering, Universiti Putra Malaysia, Serdang 43400, Malaysia

**Keywords:** biosensor, microfiber, biophotonics

## Abstract

Biosensors are central to diagnostic and medicinal applications, especially in terms of monitoring, managing illness, and public health. Microfiber-based biosensors are known to be capable of measuring both the presence and behavior of biological molecules in a highly sensitive manner. In addition, the flexibility of microfiber in supporting a variety of sensing layer designs and the integration of nanomaterials with biorecognition molecules brings immense opportunity for specificity enhancement. This review paper aims to discuss and explore different microfiber configurations by highlighting their fundamental concepts, fabrication processes, and performance as biosensors.

## 1. Introduction

With the world’s population increasing beyond 8 billion, one massive challenge is to ensure that everyone has access to proper healthcare and medicine. This focus has been highlighted as one of the Sustainable Development Goals set by the United Nation in 2015 with the expectation of complete implementation by 2030 [1]. This effort, however, has been greatly disrupted by the emergence of the COVID-19 pandemic in 2019, which recorded infections in more than 600 million people and led to 6 million deaths worldwide [2]. The disease, which transmits easily through respiratory liquid particles, highlighted the need for rapid and sensitive diagnostic tools that can assist in identifying infection and controlling its spread. Features such as quantitative detection and high specificity are also greatly desirable to allow clinical monitoring as well as the administration of proper treatment. As the world slowly progresses toward post-pandemic life, the need for high-performance diagnostic tools has not abated. Increasing population and high poverty rates mean that the next pandemic could be just around the corner. Tuberculosis and malaria are currently on the rise, while tropical countries are still subjected to the ever-present threat of dengue fever.

The optical fiber sensor has been deemed as a viable alternative with advantages such as passivity, immunity to electromagnetic interference, multiplexability, and being compact in size. The confinement of light within the micro-sized core means that minute changes to the fiber can affect signal propagation substantially and lead to observable output. Since light propagation in optical fiber is governed by the refractive index (RI), the sensing approach that relies on changes to the RI is a surefire way to produce an optical fiber sensor. The Fresnel reflection from optical fiber tips have been utilized for detection of acid, corrosion, and biofilm [3]. An optical sensing transducer can also be realized simply by exploiting the evanescent wave excited from U-bent optical fibers [4,5]. A more complex method is as demonstrated by fiber Bragg grating (FBG)—an optical fiber with periodic refractive index grating written to its core. FBG functions by detecting variations to RI or the spacing of its grating to produce shifts in the Bragg wavelength. Its capability is validated by numerous real-world deployments for the monitoring of civil structures [6].

Detection using optical fiber sensors can be made specific by introducing a sensing element that reacts solely to the target. A typical approach is to expose the core using chemical etching or tapering which creates a microfiber region that functions as the sensing area. The sensing element is then deployed onto the sensing area and the presence of the complementary target will result in RI changes that are then reflected onto the propagating light. This method has been employed for the detection of gas and chemicals. A test of 2D materials on etched-clad fiber found that graphene/graphene oxide composite exhibited excellent humidity-sensing performance [7]. In [8], the tapered optical fiber coated with a palladium/graphene oxide layer demonstrated specificity and stability toward exposure to hydrogen gas. Polishing a part of the cladding to create a fiber transducer with a D-shaped cross section also works on the same basis, with the exposed section serving as a sensing region that can be functionalized for a specific sensing purpose [9]. The same concept can be applied for biosensing purposes as well. Complementary ligands to the desired target are immobilized onto a microfiber surface which allows, the binding and creation of a new complex with different RIs [10,11]. As a result, light interacting with the modified layer will react in proportion to the RI variation that occurs on the microfiber surface. The following subsections will look into various microfiber geometries, their sensing principles, and biosensing applications.

## 2. Tapered Optical Fiber for Biosensing Applications

Tapered optical fibers were first used as fiber couplers [12]. With much success, the application of the tapered fibers continued to flourish within the communication field as beam expanders and interferometers. A few years following the tapered fiber coupler discovery, researchers exploited the interaction of the structure’s evanescent field with the external surrounding which resulted in an optical refractometer—its first application in the sensing field [13]. An optical microfiber is typically a standard optical fiber with a narrowed region that is either etched or tapered. As the behavior of light within the fiber is governed by the refractive index of the core and cladding layers, the diameter change causes a portion of the light to penetrate the external surrounding, creating an evanescent field that decays exponentially into the external medium.

### 2.1. Fabrication of Tapered Optical Fiber

**Figure 1:**
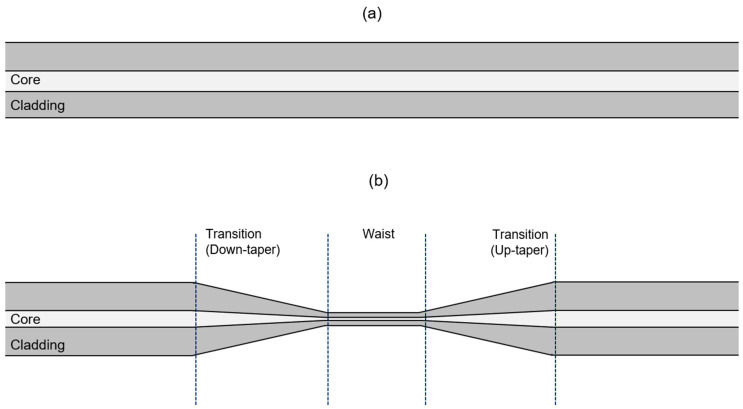
(**a**) A standard optical fiber and (**b**) a tapered fiber that has 3 distinct regions—down-taper, waist, and up-taper.

A standard optical fiber is characterized as a cylindrical strand consisting of a germanium-doped (Ge) silica core surrounded by another silica layer called the cladding (Figure 1a). As the name suggests, tapered optical fibers are generally the product of an optical fiber that has gone through a dimension alteration process, specifically a diameter narrowing modification on a particular region of the fiber, as shown in Figure 1b. Such a manipulation can be conducted through either one of the two mechanical fabrication methods: chemical etching or heat pulling.

Chemical etching is a controlled corrosion process where a corrosive agent is used to either remove or reshape a certain object. Previous studies have shown the successful usage of hydrofluoric acid as the corrosive agent to chemically etch the cladding layer of an optical fiber [14,15]. The chemical reaction is as follows [16]:

SiO_(s)_ + 3H_2_F_2(l)_ = H_2_SiF_6(l)_ + 2H_2_O_(l)_

where the reaction of silicon oxide (SiO) (the main element of the optical fiber) and hydrofluoric acid (H_2_F_2_) liquefies the optical fiber, forming a colorless liquid known as hexafluorosilicic acid (H_2_SiF_6_), making it possible to redefine the dimension of the fiber. The common practice of introducing H_2_F_2_ to the optical fiber is through the immersion of the optical fiber, or rather the region of interest, into a very high concentration of H_2_F_2_. The optimization of reaction time and mechanistic path are crucial factors in determining the resulting structure. An attractive attribute of this method is its potential to produce tapered fibers in batches. However, it takes a few hours to complete the process. As the solvent is corrosive, the optical fiber is prone to degradation and the time of removal must be precise or the fiber will be damaged. On the other hand, the rate of reaction is very dependent on the concentration of Ge within the core. In areas where the Ge is concentrated, the rate of reaction between SiO and H_2_F_2_ is slow. Thus, the method will only work should the core of the fiber have a suitable Ge profile, which most standard commercial single mode fibers lack. Due to the tedious management, chemically etched tapered fibers are often implemented during the fabrication of fiber-tip probes.

Another method that has been exploited for the same matter is heat pulling [11,17,18,19,20,21]. The basic idea is to elongate the fiber by fixing its ends to a motorized jig that pulls them in opposite directions while supplying a heat source to the area of interest. The difference between tapers fabricated through this method and those that are chemically etched can be found in the refractive index profile of the tapers. With the heat-pulling method, although the diameter may have decreased, the core/cladding interface is still preserved—hence light is guided through a core/cladding/air structure. Conversely, chemically etched tapers have their cladding layer removed completely, leaving only the core layer in the waist region. This results in the occurrence of light propagation through a core/air structure in the waist. Thus, with elements of varied refractive indices making up the core/cladding interface, tapers may develop different optical characteristics. The crucial points in this fabrication method, specifically, are to ensure that the fiber is heated uniformly and that the region is always cylindrical. These two constraints can be controlled by choosing the right heat source and proper engineering. Hoffman et al. also highlighted that a well-stripped and clean fiber prior to the taper process will yield a higher transmittance percentage [20].

Many types of heat sources have been utilized in previous studies during the fabrication process. The temperature distribution in most of them resemble a Gaussian distribution, where peak temperature is focused near the central region and decreases outwards. The flame method was among the first reported successes in creating subwavelength diameter optical fibers. However, the management of the flame’s gas flow rate and purity pose problems in terms of reproducibility and homogeneity of the taper produced, which makes it hard to control. Another alternative is replacing the flame with a carbon dioxide (CO_2_) laser [22]. This method has been reported to provide easy control over the heating region, especially in terms of length and uniformity of heat applied along the designated length. Both the laser and flame differ fundamentally from one another as the heat and radiation are absorbed within the fiber when it is heated with the CO_2_ laser, whereas the flame method radiates heat from the external surface of the fiber. Due to this, heating with CO_2_ shares an inverse square relationship with the fiber radius, thus limiting the minimum diameter it can achieve. Diameters that are less than 1 µm may be very difficult to obtain with this method. Electrical micro-furnaces can be considered as a viable option as a heat source when it comes to good control of heating uniformity. The heating temperature can be controlled easily by manipulating the current through a resistive load. As its fundamental principle of heating is similar to the flame method, this method allows sub-micron tapering with precise control of the heating temperature.

### 2.2. Classifications of Tapered Optical Fiber

Tapered fibers can be classified into two major categories: adiabatic and non-adiabatic. A taper is considered as adiabatic when the taper angle along the transition length is small enough to avoid loss of power from the fundamental mode as light propagates along the tapered region. Following this thought, adiabaticity is dependent on the relationship between the taper length, $Z_{t}$, and the beating length between two modes, $Z_{b}$. They are defined as [23]:

(1) $$z_{t}=\frac{R}{\tan(\Omega )}$$

(2) $$z_{b}=\frac{2\pi }{\beta_{1}-\beta_{2}}=\frac{\lambda }{n_{eff1}-n_{eff2}}$$

where Ω in Equation (1) is the taper angle at a particular radius (R); and in Equation (2), $\beta_{1}$ and $\beta_{2}$ are the propagation constants for the fundamental mode and the first excited mode that is symmetrical to the fundamental mode, respectively, at R; and while λ is the wavelength propagating at that point, $n_{eff1}$ is the effective refractive index of the fiber and $n_{eff2}$ is the effective refractive index of the core/clad/air interface. To be adiabatic, $Z_{t}$ must exceed $Z_{b}$ [23]. As a result, the main portion of power resides within the fundamental mode (LP_01_) and coupling with high order modes does not occur.

On the other hand, non-adiabatic tapered fibers have abrupt transition angles and consequently, short taper lengths. This allows LP_01_ to penetrate the core/clad/air interface, propagate, and couple with higher order modes along the tapered region, thus creating a boundary of evanescent waves. Although in certain applications these losses are not favored, strong evanescent fields are very beneficial for optical sensing and spectroscopy.

### 2.3. Generation of Evanescent Waves on the Surface of a Tapered Optical Fiber

**Figure 2:**
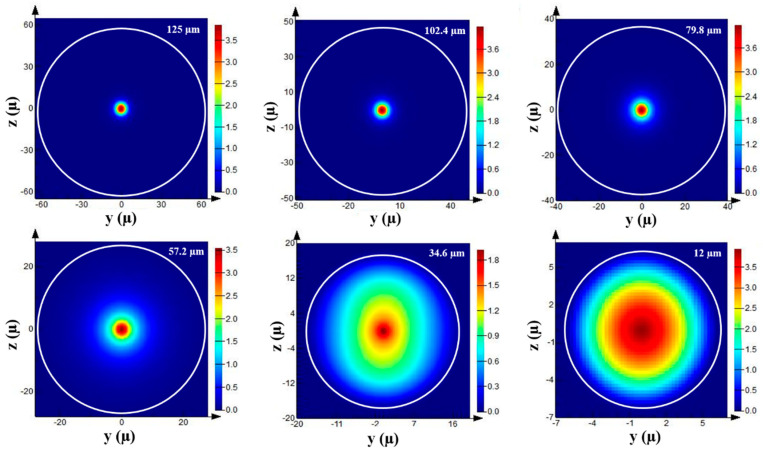
Modal evolution during transition from non−tapered region to taper waist (reprinted with permission from [25]).

Excited modes that penetrate the core/cladding/air interface and continue propagating within the external surrounding are known as evanescent waves. The boundaries of the evanescent waves that reflect the distance of its existence beyond the core/cladding/air interface is known as the penetration depth. It is described mathematically as [24]:

(3) $$E\left(x\right)=E_{0}{}^{\left(\frac{-x}{d_{p}}\right)}$$

where *x* is the distance from the core/cladding/air interface, $E_{0}$ is the magnitude of the field, and $d_{p}$ is the penetration depth, which is further defined as [24]:

(4) $$d_{p}=\frac{\lambda }{2\pi \sqrt{n_{co}^{2}\sin^{2}\theta -n_{cl}^{2}}}$$

where *λ* is the wavelength of the light source, $n_{co}$ and $n_{cl}$ are the refractive indices of the core and cladding, and θ is the incident angle. Figure 2 is a simulation acquired using Lumerical Mode Solutions software to map the H-field evolution along a tapered optical fiber [25]. Strong confinement of the Hy field can be observed in the non-tapered region. Nevertheless, the narrowing of the diameter induced the modes to excite and penetrate the fiber/external surrounding interface. Hence, the extension of Hy field propagation beyond the core region was observed. Due to the sensitivity of the evanescent waves toward changes in the environment, the principle has been used to detect changes in the external medium by measuring wavelength shift, absorption, reflection, and fluorescence.

Wavelength shift-based biosensors operate similarly to a Mach–Zehnder interferometer [11,26,27,28,29]. The up-taper region of the tapered fiber promotes the coupling of light modes in the external medium and the fundamental mode which would cause an interference. In this case, light transmission is defined by [17]:

(5) $$I=I_{1}+I_{2}+2\sqrt{I_{1}I_{2}}\cos(\Delta \Phi )$$

where *I* is the intensity of output in total, *I*_1_ and *I*_2_ are intensities of guided and unguided modes, and ΔΦ is the phase difference which is affected by the difference between the effective refractive index of the core and the cladding along the waist length. Thus, an increase of RI in the external surrounding will reflect as a red shift, or peak shifting toward the longer wavelength region.

Taper geometry plays a contributing role in the efficiency of the sensor which is determined based on transmittance efficiency and free spectral range of an individual fringing peak. Transmittance efficiency is directly proportional to the penetration depth of the evanescent wave created on the surface of the tapered optical fiber. This will affect the interaction of evanescent waves with the external surrounding and sensor sensitivity. On top of that, transmittance efficiency is particularly important for absorbance- and intensity-based sensors as it determines the operational range for the sensors. Wavelength shift-based sensors, on the other hand, prioritize wavelength peak and range over intensity. Hence the free spectral range (FSR) of the taper peaks play a huge part in determining the operational range of the sensor rather than transmittance efficiency. Both FSR and transmittance efficiency highly depend on the parameters that make up the geometry of the taper: taper angle, waist length, and waist diameter. In [17], it has been reported that smaller waist diameter contributes to an abrupt taper angle which would lead to more interference. More interference will produce fringes with shorter FSR and narrow full width at half maximum (FWHM) that may benefit free-label sensors for biomolecules with low molecular weight at very low concentrations. However, this may not be ideal for the detection of high molecular weight molecules as the wavelength shift is predicted to be larger, which may exceed the short FSR causing peak overlaps.

When implemented in a biosensor, the wavelength shift corresponds to the presence and quantity of the targeted biological molecule. However, the correlation between the wavelength shift and the concentration of the targeted biological molecule is only valid provided that the sensor is selective toward the targeted biological molecule. To ensure selectivity, bio-recognition molecules that are complementary to the target are immobilized on the surface of the microfiber. Bio-recognition molecules that have been used on tapered fiber-based sensors can be enzymes, antibodies, aptamers, and complementary DNA, to name a few. The principle behind this technique is to create a specific affinity between the tapered fiber sensor and the target so that the targeted molecule will bind to the surface of the sensor and create the refractive index change within the external surrounding of the sensor which will be reflected as a wavelength shift. The most common type of bio-recognition molecule used on tapered fiber sensors are antibodies. Mustapha Kamil et al. have reported the use of anti-DENV II E protein antibodies as the bio-recognition molecule on a dengue sensor that was targeting dengue virus type II E proteins [26]. Prior to immobilizing the antibodies, the surface of the tapered optical fiber was introduced to sodium hydroxide in order to decorate the surface with hydroxyl groups. Next, silanization with (3-Aminopropyl)triethoxysilane (APTES) was conducted that would bridge the inorganic silica surface with biological molecules. Following silanization was the activation with glutaraldehyde and antibody immobilization. The study reported good selectivity in a sample with avidin and yielded a spike recovery percentage between 83% to 89%. Consequently, antibodies were used in the following research work pertaining to dengue sensors as well [25,30,31].

**Figure 3:**
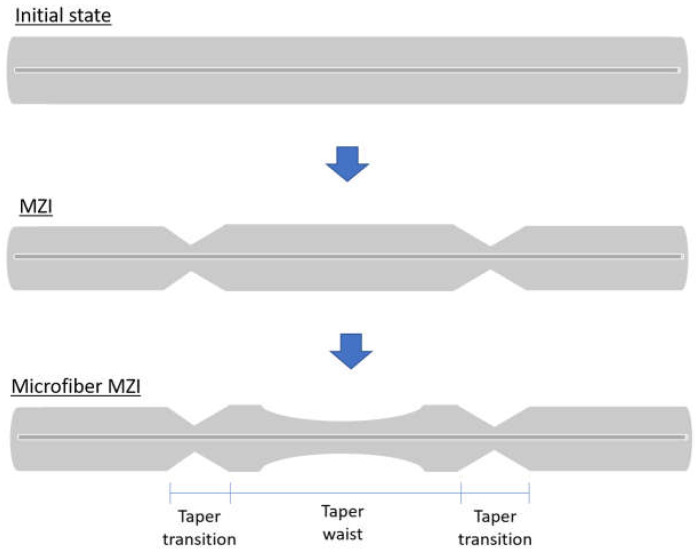
Dual taper sensor preparation as reported in [32].

Different tapered fiber structures were reported to be used as sensors, too. For example, Li et al. reported a wavelength shift-based biosensor using a dual-taper as a transducer to detect Staphylococcus aureus [32]. The configuration of the transducer is shown in Figure 3. It consisted of an adiabatic taper sandwiched between two non-adiabatic tapers. The role of the first taper was to excite the cladding modes while the adiabatic taper in the middle sustained the propagation of the excited modes on its surface as evanescent waves at different intensities. As these evanescent waves reach the third taper, modes recoupled which created a similar fringing pattern such as a standard non-adiabatic taper. To enhance the specificity of the sensor, anti-Staphylococcus aureus IgG antibodies were functionalized on the surface of the taper waist. With a taper diameter of 10.2 µm, the limit of detection achieved was as low as 11 CFU/mL with a detection response time of less than 30 min. For further improvement on the detection limit, Ling Chen et al. proposed narrowing down the full width at half maximum (FWHM) value of the spectrum [33]. To achieve a narrow FWHM, the tapered fiber sensor was deployed in a fiber ring laser setup. Equations (6) and (7) elaborate the relationship between FWHM and detection limit:

(6) $$Limit\;of\;detection=\frac{Resolution}{Sensitivity}=\frac{\sqrt[{3}]{\sigma_{ampl-noise}^{2}+\sigma_{temp-induced}^{2}+\sigma_{spect-res}^{2}\;}}{Sensitivity}$$

where

(7) $$\sigma_{ampl-noise}^{2}\;\approx \;\frac{FHWM}{\left(4.5\right){\left(SNR\right)}^{0.25}}$$

The same group also proposed the use of a no-core tapered fiber as means of improving the durability of the sensor. The large refractive index difference between the no-core tapered fiber and the external surrounding still drives the excitation of modes and the creation of evanescent waves—without the need for ultrashort waist diameters (below 10 µm). A no-core based tapered fiber sensor was also investigated for the detection of glucose [34]. Similar to the operation reported by [33], the no-core fiber was spliced in between two single-mode fibers in order to induce the excitation of modes from the core mismatch and the large refractive index difference between the no-core fiber and the external surrounding. Specificity toward glucose was ensured by immobilizing glucose oxidase as the bio-recognition molecule, which was facilitated by deposited graphene oxide on the surface of the fiber. The proposed sensor achieved a sensitivity value of 0.0321 nm/(g/L) for glucose. The authors also investigated simultaneous temperature sensing on the same sensor. Using a measurement matrix, the two parameters, glucose concentration and temperature, were successfully distinguished and calculated.

Tapered optical fibers can also support other modes of measurement aside from wavelength shifts. One example is the implementation of localized surface plasmon resonance (LSPR) using a gold-coated tapered fiber sensor [35,36,37]. The principle of LSPR is the utilization of interactions between conduction electrons from deposited metal nanoparticles with the incident light, or in this case evanescent waves. A difference in the refractive index will be reflected in a measurable shift of the peak absorbance wavelength. In the reported work, the LSPR sensor was used for the detection of the amino acid taurine. The bio-recognition molecule used in the work by Sharma et al. was an enzyme called taurine dioxygenase [35]. The key role of the enzyme was to facilitate the reaction of taurine, alpha-ketoglutarate, and oxygen to yield sulphite, succinate, and amino-acetaldehyde which would lead to a change in the refractive index. A change of 0.0115 in absorbance is obtained for every 1 mM change of taurine concentration with a detection limit of 53 µM. Other than LSPR, material deposition on the surface of the tapered optical fiber may also enhance other sensing properties such as sensitivity and detection limit by increasing surface area, providing more active sites for immobilization of biorecognition molecules or/and targets, increasing fiber affinity toward the target, or amplifying the evanescent field. The materials that have been reported in recent studies include zeolitic imidazolate [38], pyrole/poly(vinyl-alcohol)–glucose oxidase [39], and poly(phenylboronic acid) [40]. Applications related to evanescent wave-based biosensors involving tapered optical fibers are listed in Table 1 with their sensing parameters.

## 3. Microfiber Bragg Gratings for Biosensing Applications

**Figure 4:**
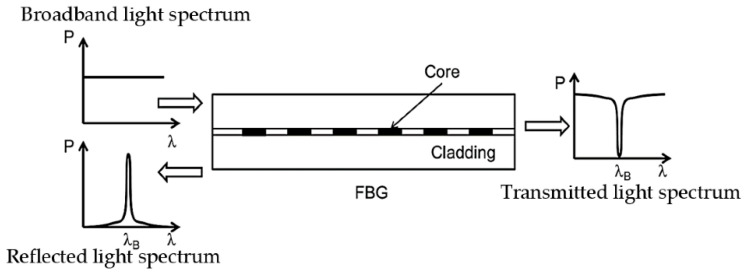
Basic principle of FBG [43].

Fiber Bragg gratings (FBGs) are repeated micro-inscriptions of a few millimeters in length within a single-mode fiber. These inscriptions are created by exposing the core of the fiber to a periodic pattern of intense light which would permanently increase the refractive index of the core and create a fixed index modulation according to the exposure pattern. As shown in Figure 4, a narrow spectrum is reflected due to the gratings while the remaining portion continues propagating forward. The reflection spectrum peaks at the Bragg wavelength $\lambda_{B}$, which is defined as:

(8) $$\lambda_{B}=2n_{eff}\Lambda$$

where $n_{eff}$ is the effective refractive index and *Λ* is the period of grating. The operation of an FBG sensor typically exploits the influence of $n_{eff}$ toward $\lambda_{B}$. In other words, any changes to the FBG that affects $n_{eff}$ will be measurable in terms of Bragg wavelength shift. For long-period FBGs, period gratings are usually between 100–700 µm while period gratings in short-period FBGs are smaller by hundreds of nanometers. With a cladded-FBG, it is intrinsically sensitive toward temperature and axial strain as the Bragg resonance is restricted to react with light confined within the fiber only. Nevertheless, other FBG geometry variations have been proposed to eliminate this limitation. Among them is microfiber-based Bragg gratings (MFBGs), where the diameter of the FBG has been shortened or modified in order to produce leaky modes that are able to interact with the external medium surrounding the FBG.

An example is an unclad uniform FBG, also known as a bare FBG, where the core is exposed to the external surrounding medium [44]. Having the core exposed is advantageous, especially for detecting biological molecules. De Lisa et al. was the first to report the detection of Escherichia coli (E. coli) antibodies using a long-period FBG that was functionalized with an anti-human E. coli IgG antibody [45]. Operational range was between 2–100 µg mL^−1^ with a peak signal and flow rate at 40 min and 250 µL min^−1^, respectively. In 2006, the LPG FBG configuration was modified with self-assembled gold colloids on the core surface. For the detection of anti-dinitrophenyl, the sensor achieved a detection limit of 9.5 × 10^−10^ M [46].

**Figure 5:**
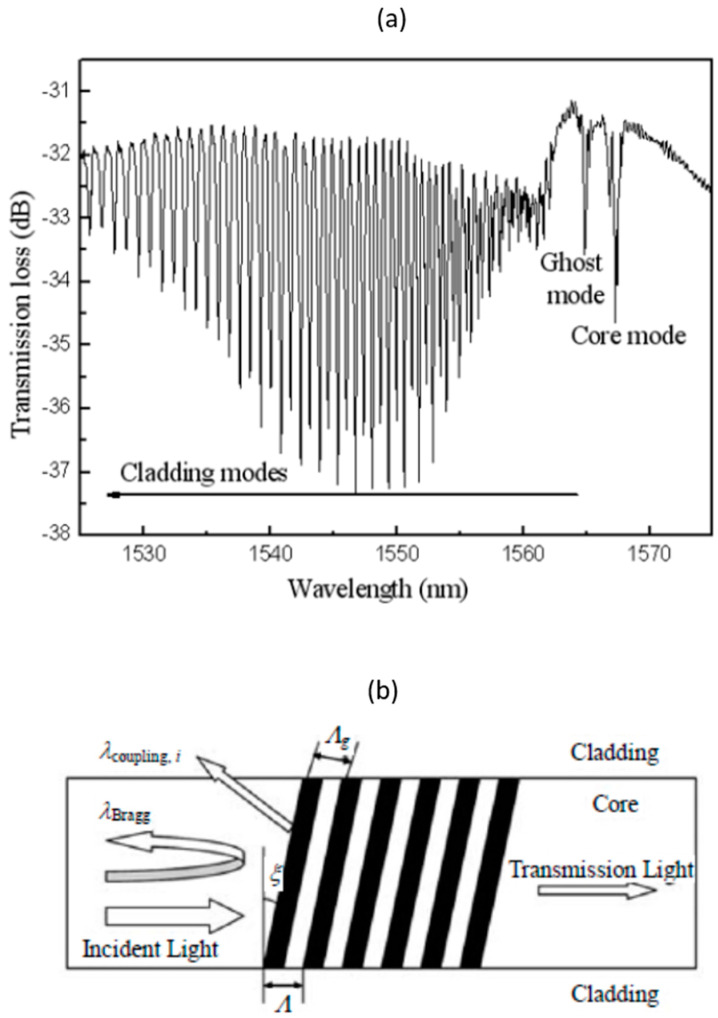
(**a**) Transmission spectrum of tilted−FBG and (**b**) schematic diagram of the light reflection in tilted−FBG [47].

Another geometrical MFBG variation is tilted-FBG (TFBG) which has a similar periodic refractive index modulation as a normal FBG. The only difference is that the gratings are angled (Figure 5b) which will lead to the redirection of some light to the cladding and more complex mode coupling. This would result in a transmission spectrum with many resonance peaks as shown in Figure 5a. The relationship between coupling wavelengths for a given cladding mode and its effective refractive index is defined as:

(9) $$\lambda_{Bragg}=(n_{eff,\;core\;}+n_{eff,\;clad})\Lambda_{g}$$

Each resonance peak is a result of the core mode coupling with a group of backward-propagating cladding modes. This means that the positions of these peaks depend on the effective refractive index of the cladding mode which is influenced by the medium surrounding the cladding surface—which makes it feasible for refractometry measurement, either within the external medium or in a thin coating deposited on the fiber’s surface.

### Fabrication of MFBG

**Figure 6:**
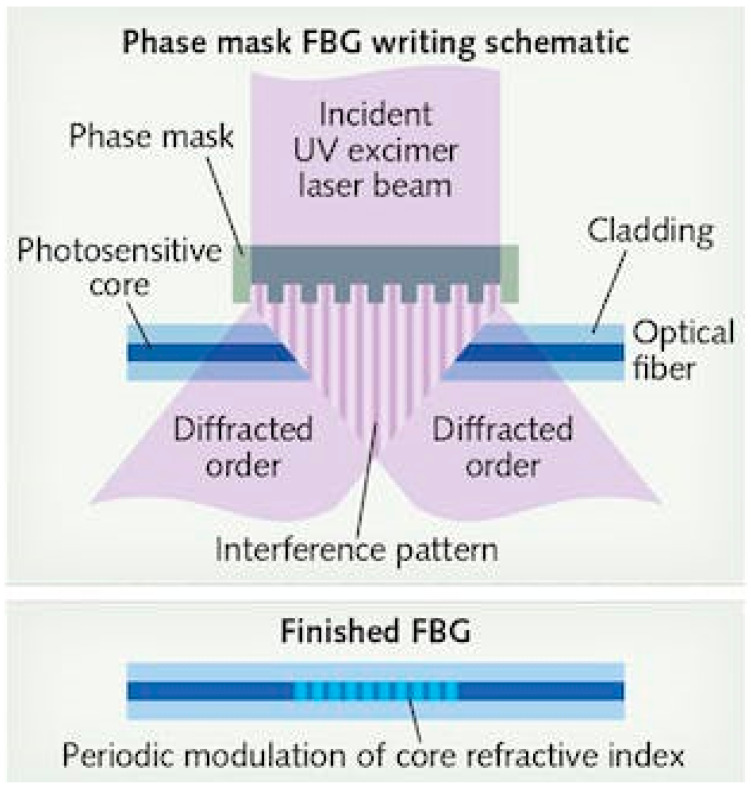
Fabrication process of FBG [48].

The gratings in the fiber were firstly fabricated using internal writing and the holographic technique. The holographic technique involves the overlapping of two UV light beams that would generate a periodic interference pattern with an equivalent periodic index grating. Among the notable advantages of using this method is the ability of imprinting gratings without removing the glass cladding. In addition, the period depends on the angle between the two interfering UV light beams. Hence, the gratings could be made to function at longer wavelengths. In the early 1990s, the phase mask technique (Figure 6) became trendy and favored due to its simplicity and flexibility. Unlike the holographic system, fiber alignment for the phase mask technique is easier and gratings can be fabricated with controlled spectral response characteristics. With the phase mask technique, the output of an excimer laser is directed through a phase mask which diffracts a laser beam into various orders. This causes overlaps and interference between beams in the mask vicinity. The interference creates a periodic laser intensity modulation that would result in the gratings on the core of the fiber, as shown in Figure 6.

**Figure 7:**
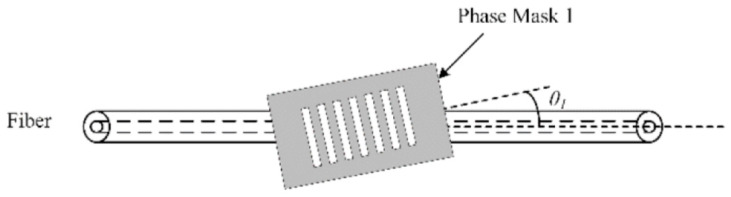
Fabrication process of tilted-FBG [49].

To fabricate TFBG, the phase mask technique is also used where a UV light is shone through a photo mask onto a bare fiber. The difference with TFBG is the alignment of the photomask where the planes are written with a tilt that is relative to the longitudinal axis of the fiber (Figure 7). The degree of tilt influences the selection of cladding modes that will be excited. Hence, the operating range of a TFBG sensor can be manipulated by the degree of tilt.

For MFBG, the removal of the cladding layer can be achieved via etching with a very strong acid, such as hydrofluoric acid or side-polishing, that will have similar geometries to a D-shaped fiber [50]. To etch, the acrylate buffer coating of the optical fiber is removed first prior to dipping into 48–52% of hydrofluoric acid. The degree of etching depends on the rate of length/time. After obtaining the desired design, the etched area is rinsed using methanol and dried. Conversely, side-polishing can be achieved using a motor-driven polishing wheel [50].

MFBGs have been among the various optical fiber biosensor designs to be employed extensively. Like tapered optical fiber biosensors, the key component in MFBG-based biosensors is the development and functionalization of the active sensing layer on the surface of the fiber. Marques et al. employed the principles of LSPR on an MFBG-based biosensor for the detection of streptavidin [51]. The bio-recognition molecule used was biotin which was immobilized on gold nanoparticles that were electrostatically assembled on the surface of the MFBG facilitated by a poly(allylamine hydrochloride)(PAH) polycation layer. The detection limit was as low as 19 pg/mm^2^, which was a notable achievement. On the other hand, Bekmurzayeva et al. opted for a transmittance wavelength shift in their MFBG-based thrombin sensor instead of LSPR [52]. Alternatively, silanization and activation were performed on the etched surface of the MFBG prior to the immobilization of the bio-recognition molecules, which in this case were thrombin-binding aptamers. This was decided in order to avoid the deposition of metal nanoparticles (which is a complicated and expensive fabrication process). The MFBG sensor managed an operation range of 10 nM to 80 nM. A DNA sensor using MFBG was also reported using reduced-graphene oxide as the active sensing layer on the etched surface of the MFBG [53]. Although no bio-recognition molecules were used in this experiment, the authors monitored the absorbance spectrum at a wavelength that was specific to the targeted double-stranded DNA. The sensor was highly sensitive with a detection limit of 261.87 pg/μL.

Aside from MFBGs, TFBGs have been reported as biosensors, too. Guo et al. developed an SPR-based TFBG sensor embedded in a microfluidic channel for urinary proteins [54]. The generation of surface plasmons were induced by an ultra-thin silver film that was coated on the surface of the TFBG. The sensor configuration attained a sensitivity value of 5.5 dB/(mg/mL) and a detection limit of 1.5 × 10^−3^ mg/mL. Similar configurations using gold were also reported for real-time cellular monitoring. More specifically, the authors were monitoring the effects of trypsin, serum, and sodium-azide toward cells that were attached on the surface of the TFBG with the aid of fibronectin (a known cell-adhesive). The detachment of cells will affect the refractive index surrounding the TFBG sensor, which would reflect in a resonance peak shift. MFBG- and TFBG-based biosensors are presented in Table 2 along with their notable performance indicators: mode of detection, targeted molecule, biorecognition molecule, detection limit, and resolution.

## 4. D-Shaped Fiber for Biosensing Applications

**Figure 8:**
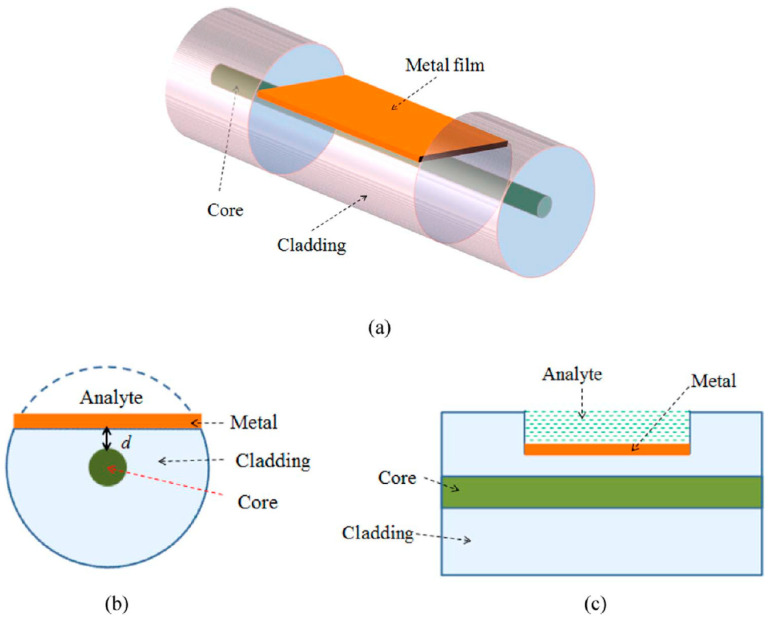
(**a**) D-shaped fiber coated with a metallic film; (**b**) cross section view; and (**c**) schematic of D-Shaped fiber [58].

D-shaped fiber is generally a standard optical fiber that has been side-polished to attain a flat side which eventually forms a “D” shape from a cross-sectional point of view. It functions similarly to a tapered optical fiber, where evanescent waves generated on the flat surface are vulnerable to changes of effective refractive index and energy distribution within the external surrounding. However, a bare D-shaped fiber has been shown to possess relatively low sensitivity. To improve the sensing performance, Wang et al. improvised the configuration by adding gold film onto the flat surface [57]. Better sensitivity was achieved with a 10% increment in reflectivity. After their success, other investigations were carried by testing different types of fibers coated with other materials. In Figure 8, the basic structure of a D-shaped fiber is shown with the deposition of a metal film.

Changes in the external surrounding can be detected and measured based on the optical wavelength shift. As light propagates through the fiber, internal reflections will occur that would result in the generation of evanescent waves on the flat surface of the D-shaped fiber. These waves will interact with plasma waves on the metal film where the Fresnel coefficient can be defined as [58]:

(10) $$r_{ij}=\frac{n_{i}^{2}k_{jy}-n_{j}^{2}k_{iy}}{n_{i}^{2}k_{jy}+n_{j}^{2}k_{iy}}$$

where $k_{iy}=\frac{\omega }{c}\sqrt{n_{i}^{2}-n_{i}^{2}sin^{2}\theta },\;i=1,\;2,\;3\;\mathrm{and}\;j=i+1$ for any two adjacent mediums while θ is the incident angle. From the two equations, the refractive index has a significant effect on the intensity of the optical reflection. Hence, changes in the external surrounding will be reflected as an intensity change in the resonance wavelength.

### Fabrication of D-Shaped Fiber

The conventional ways of fabricating D-shaped fibers are chemical etching and mechanical polishing. For mechanical polishing, the optical fiber to be polished is fixed into a curved V groove which has been customized to fit the specified fiber diameter and intended polishing depth. Once fixed, the cladding is polished away with a limited length of several millimeters [50]. Another polishing method that is being practiced to fabricate D-shaped fibers is the wheel polishing technique, which has better flexibility in terms of polishing distance. Coarse abrasives will be used first to remove large parts of the cladding, which will be followed by a fine abrasive to reduce scratches.

The other alternative in D-shaped fiber fabrication is chemical etching. The standard solution used to etch the fiber is hydrofluoric acid [50]. The etching rate in this case depends on the concentration of the acid, temperature, and also the configuration of the optical fiber that is intended to be etched. In addition, as the product of the reaction is water, the concentration of H_2_F_2_ is very likely to change over time, which will also affect the etching rate. Aside from water, bubbles and sols will also form from the reaction and will adhere onto the fiber’s surface. This is an unwanted phenomenon as the precipitate will slow down the etching rate, leading to a rough and brittle surface.

**Figure 9:**
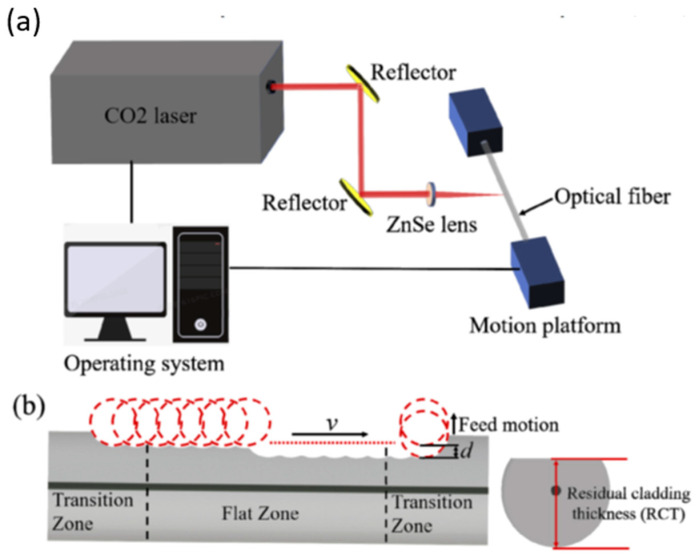
(**a**) Schematic diagram of the D-shaped fiber fabrication device and (**b**) the cross-section of the D-shaped fiber [50].

Recently, Hu et al. reported the fabrication of D-shaped fibers using a pulse CO_2_ laser. The setup used for the fabrication is shown in Figure 9. First, the laser focal plane was focused on the top surface of the fiber. Next, the fiber was moved front-to-back along its axis while feeding it up slowly. There will be three regions on the processed fiber: two transition zones and a flat zone in the middle.

In the past couple of years, researchers have been particularly interested in realizing the principles of SPR sensing of D-shaped fibers. Cennamo et al. utilized a D-shaped plastic optical fiber for the detection of thrombin in a nanomolar range as a clinical marker for blood coagulation and homeostasis [59]. The selectivity of the sensor was ensured by immobilizing specific aptamers that are complementary to thrombin—thrombin binding aptamer (TBA). Prior to the immobilization of TBA, the gold layer was modified with thiolated-polyethylene glycol (PEG), 6,8-dithio-octanoic-PEG, and biotinylated-PEG. The range operation was within the nanomolar regime with a short analysis time of 5 to 10 min. Two years after the aforementioned work, the same research group used similar sensor configurations for the detection of the SARS-CoV-2 S1 spike protein [60]. The biorecognition molecule used in this case was an aptamer complementary to the receptor-binding domain of the SARS-CoV-2 S1 spike protein. The detection limit obtained was as low as 37 nM, of which selectivity was successfully assessed using bovine serum albumin, AH1N1 hemagglutinin protein, and the MERS spike protein. Recent literature on D-shaped fiber-based biosensors is presented in Table 3 along with their notable performance indicators: mode of detection, targeted molecule, biorecognition molecule, detection limit, and resolution.

## 5. Conclusions and Future Outlook

The ultimate objective of this review was to cover in-depth the fundamental concepts of the sensing solutions provided by microfiber-based biosensors, innovative fabrication techniques, and trending biosensing applications pertaining to microfibers. It is without a shred of doubt that microfibers have shown exceptional traits and are very advantageous to have in a sensor. These include its compact size, high-fractional evanescent fields, high sensitivity and selectivity, adaptability, and flexibility in terms of sensor design. However, their practicality and implementation in a real-world setting have yet to be realized. This is mainly due to some limitations of microfiber sensors that have yet to be solved.

The first glaring limitation is the fragility of the microfibers. Narrowing down the waist diameter puts a high cost on the strength of the microfiber that has brought difficulties during fabrication and installation into any real systems. In recent years, researchers have proposed a number of ways to enhance the durability of microfiber sensors. As reviewed in this paper, nanomaterials such as graphene, gold, and polymers have been used to increase the integrity of sensors [36,63,64]. The integration of no-core fiber was also proposed in a number of studies in order to create the core mismatch that will induce the excitation of modes without having to reduce the waist diameter of the sensor [33,34]. Another limitation is the inability to multiplex like its counterpart, the FBG sensors. The multiplexing capacity of FBG sensors has allowed easy integration of simultaneous sensing which is still a puzzle for optical microfiber sensors. We have come across some feasible solutions in recent literature. One of the ways was to use an algorithm to distinguish signals from different targets [34]. Another probable solution is to integrate multiple tapers in one system—which has yet to be extensively reported on. We do foresee that sieving through data and analysis of data may be challenging with multiple tapers. However, with the integration of machine learning, this may be solved.

As a future outlook, studies in optical microfiber sensors may be extended toward simultaneous sensing of multiple parameters, long term stability, improving durability for integration into real-world devices, and integration with machine learning. Efforts previously mentioned, although they may not be the complete answer to solving the limitations, have brought us a step forward in improving the robustness of microfiber sensors and should be continued so that the advantage of optical microfiber sensors can be fully exploited.

## Figures and Tables

**Table 1:** Tapered optical fiber-based biosensors.

Targeted Analyte	Sensing Layer	Sensitivity	Limit of Detection	Ref.
Uricase	Graphene oxide	0.0089 nm/µM	259 µM	[36]
Staphylococcus	Dual-taper	-	11 CFU/mL	[32]
Taurine dioxygenase	Gold nanoparticles	0.0190 AU/mM	53 µM	[35]
Urease	polyaniline-zinc oxide	-	10 nM	[27]
Lipase	Zeolitic imidazolate framework	0.9 nm/nM	0.23 nM	[38]
Glucose	Gold nanoparticles	0.9261 nm/mM	322 µM	[37]
Glucose	pyrrole/poly(vinyl alcohol)-glucose oxidase	8.7 × 10^−3^ µWmM^−1^	-	[39]
Glucose	graphene oxide + gold nanoparticles	1.06 nm/mM	2.26 mM	[34]
Glucose	poly (phenylboronic acid)	0.1787%/nM	5 mM	[41]
Listeria monocytogenes	tapered single more no core fiber	-	1.0 cell/mL	[33]
Dengue E protein	Anti-Dengue E antibody	5.02 nm/nM	1 pM	[25]
Dengue E protein	PAMAM	19.53 nm/nM	1 pM	[30]
Dengue E protein	Graphene oxide	12.77 nm/nM	1 pM	[28]
Dengue E protein	PAMAM + Graphene oxide	13.25 nm/nM	1 pM	[31]
Avidin	Biotin	20.368 nm/μM	-	[42]

**Table 2:** MFBG-based biosensors.

Targeted Analyte	Sensing Layer	Grating Architecture	Sensor Performance	Ref.
Thrombin	Thrombin-linking aptamers	Etched fiber Bragg grating	LOD: 10 nM	[52]
DNA	Graphene oxide	Etched fiber Bragg grating	LOD: 261.87 pg/uL	[53]
Streptavidin	Gold nanomaterials modified with biotin	Etched long period fiber Bragg grating	LOD: 6.88 nm/(ng/mm^2^)	[51]
Proteinuria in rat urine	Gold layers of different thickness	Tilted fiber Bragg grating	LOD: 1.5 × 10^−3^ mg/mL	[54]
Cellular behavior	Gold layer + fibronectin	Tilted fiber Bragg grating	-	[55]
Aquaporin-2	Gold layer + aquaporin-2-antibodies	Tilted fiber Bragg grating	1.5 ng/mL	[56]

**Table 3:** D-shaped fiber-based biosensors.

Targeted Analyte	Sensing Layer	Sensitivity	Limit of Detection	Ref.
Bovine serum albumin	anti-BSA	1200 nm/RIU	-	[61]
Cortisol	Gold + anti-cortisol antibody	0.02 nm/log(ng/mL)	1.46 ng/mL	[62]
SARS-CoV-2 S1	Aptamer	-	37 nM	[60]
Thrombin	Aptamer	-	1 nM	[59]

## Data Availability

Data sharing not applicable.
